# Comparisons of periventricular device closure, conventional surgical repair, and transcatheter device closure in patients with congenital ventricular septal defects: A Protocol for Systematic Review

**DOI:** 10.1097/MD.0000000000018901

**Published:** 2020-01-24

**Authors:** Dongxu Li, Zhao Zhang, Mengsi Li

**Affiliations:** a Department of Cardiovascular Surgery; b Department of Anesthesiology, West China Hospital, Sichuan University, Chengdu, PR China.

**Keywords:** conventional surgical repair, network meta-analysis, perventricular device closure, transcatheter device closure, ventricular septal defect (VSD)

## Abstract

**Background:**

Surgical treatments for ventricular septal defects (VSDs) mainly include 3 approaches: conventional surgical repair (CSR), transcatheter device closure (TDC), and perventricualr device closure (PDC). PDC has been introduced in over 10 years, although there remain concerns about adverse events. This network meta-analysis is to compare PDC, CSR, and TDC in the treatment of congenital VSD.

**Methods:**

We will search MEDLINE, EMBASE, Clinical Trials, Cochrane Library, and China National Knowledge Infrastructure databases for comparative studies on device closure and conventional repair for congenital VSDs to August 2019. A network meta-analysis with frequentist frame will be performed to compare the 3 approaches involving the success rate, in-hospital indexes, and incidences of complications, using risk ratio with 95% confidence intervals.

**Results:**

This study will be submitted to a peer-reviewed journal for publication.

**Conclusion:**

This network meta-analysis will assess the safety and efficacy of PDC, CSR, and TDC in the treatment of congenital VSDs, and provide more evidence-based guidance in clinical practice.

**PROSPERO registration number:**

CRD42019125257.

## Abstract 2

Supplemental Digital Content is available in the text

## 1 Introduction

Isolated ventricular septal defects (VSDs) account for approximately 20% to 30% of all congenital heart diseases, and they are among the most common congenital heart defects.^[1,2]^ Since Lillehei et al first introduced surgical repair for VSD closure, this procedure was considered the gold standard for most VSDs.^[3,4]^ However, this method has been challenged by percutaneous transcatheter device closure (TDC) with regard to original surgical trauma, morbidity, and mortality.^[5]^ Additionally, many studies have confirmed the superior clinical outcomes and economic benefits of percutaneous closure compared with conventional surgical repair (CSR) in selected patients.^[6–9]^

Although transcatheter occlusion is minimally invasive and effective, it has several undesirable aspects: the vascular limitation, limited manipulation, and radiation.^[10]^ Hence, perventricular device closure (PDC) had been introduced in a baby by Amin et al after animal experiments.^[11]^ Subsequently, more cardiac surgeons attempted PDC in patients with congenital VSDs and reported the experiences and outcomes in their centers.^[12,13]^ However, with implantation of the metallic occluder device in the membranous septum in a VSD, the risks of device dislocation, valvular regurgitation, and heart block existed.^[14]^ The results of device closure for congenital VSDs have always been controversial.^[5,14]^ Additionally, results of comparisons among the 3 approaches (TDC, CSR, and PDC) are unclear.

Therefore, we aimed to conduct a network meta-analysis involving direct and indirect comparisons of the 3 approaches to evaluate efficacy and safety. We believe that the findings of this study will provide information for clinical strategies.

## 2 Methods

### 2.1 Study registration

This protocol is conducted according to the preferred reporting items for systematic review and meta-analysis protocols (PRISMA-P) statement.^[15]^ This network meta-analysis will be conducted according to the PRISMA extension statement.^[16]^ This protocol has been registered in the PROSPERO network (registration number: CRD42019125257).

### 2.2 Ethics and dissemination

#### 2.2.1 Ethics issues

The network meta-analysis does not require ethical approval because the original data are anonymous, which no privacy will be involved.

#### 2.2.2 Publication plan

This network meta-analysis will be published in a peer-reviewed journal after completed.

### 2.3 Eligibility criteria

#### 2.3.1 Types of studies

Randomized controlled trials (RCTs) or cohort studies are selected using the following inclusion criteria:

(1) 2- or 3-arm studies that reported at least 2 approaches among CSR, TDC, and PDC;
(2) studies that described at least 1 variable defined as follows:
  1. procedural success, with or without reasons for failures,
  2. complications, including residual shunt, arrhythmias, new-onset valvular insufficiency, pericardial effusion, incision complication, reoperation for any reasons, and death, and
  3. outcomes of follow-up patients.

#### 2.3.2 Types of participants

Patients with congenital VSDs (patients with only doubly committed subarterial VSD or acquired VSD following myocardial infarction or trauma are excluded).

#### 2.3.3 Types of interventions and comparators

The treatment group will be treated with PDC. The control group will be treated with CSR or TDC.

#### 2.3.4 Types of outcome measures

The primary outcome is procedural success rate measured in hospital. The secondary outcomes are complications, including residual shunt, arrhythmias, new-onset valvular insufficiency, pericardial effusion, incision complication, reoperation for any reasons, and death counted in hospital or out of hospital, and follow-up data.

### 2.4 Search strategy

Systematic literature searches of the MEDLINE, EMBASE, Clinical Trials, Cochrane Library, and China National Knowledge Infrastructure are conducted to identify relevant studies published up to August 30, 2019 in English and Chinese. The detailed search strategies involving treatments of VSDs are shown in Supplemental Digital Content (Supplementary File 1, http://links.lww.com/MD/D655).

### 2.5 Data collection and analysis

#### 2.5.1 Data management

The studies selected from all the databases will be integrated into Endnote X7 (Thomson Reuters, Canada). A pilot-test will be conducted to ensure the inter-rater is reliability between the reviewers before the literature selection.

#### 2.5.2 Selection process

Two independent researchers (DXL, ZZ) will conduct a systematic search on above 5 databases according to the predetermined search strategy. In the case of the abovementioned screening of documents and the extraction of data, if there is a disagreement, it will be resolved through discussion or assistance to a third reviewer. All the study selection process will be revealed in a flow diagram in accordance with the PRISMA guidelines.^[16]^

#### 2.5.3 Data extraction process

Two reviewers (DXL, ZZ) will assess the full text of the included studies. All data will be extracted independently by 2 authors (DXL, ZZ). Where available, the data included characteristics of studies, patient baselines, perioperative parameters involving procedural success rate, main complications, and follow-up data. Successful implantation is defined by correct device placement at a satisfactory position as confirmed on imaging. All devices that had to be explanted are considered unsuccessful in this study. Residual shunts included all color jets seen across the VSD after device placement. Arrhythmias included right bundle branch block, second- or third-degree atrioventricular blocks. Valvular lesions included device-related aortic or tricuspid regurgitation with exclusion of transient early lesions that disappeared in the post-deployment period.

### 2.6 Level of evidence

The level of evidence of the included 2-arm studies will be categorized according to the criteria of the Center for Evidence-Based Medicine in Oxford, United Kingdom.^[17]^ Studies achieving a score of ≥3b are considered to be of high quality.

### 2.7 Assessment of risk of bias in included studies

The methodological quality of the included RCTs will be assessed by 2 authors (DXL, ZZ) using Cochrane Risk of Bias tool, which include selection bias (random sequence generation and allocation concealment), performance bias, attrition bias, detection bias, reporting bias, and other possible bias of all the included original studies.^[18]^ And non-RCTs are evaluated by the Newcastle–Ottawa Quality scale, in which a high-quality study is defined as a study with ≥6 scores.^[19]^

### 2.8 Statistical analysis

A network meta-analysis involving a comparison among PDC, CSR, and TDC will be performed with risk ratio and 95% confidential intervals under the random effects model.^[20]^ All statistical evaluations will be performed assuming a 2-sided test at 5% level of significance, using Stata software (version 14.0; Stata Corp., College Station, TX) with “network” command.^[21]^ Consistency and inconsistency test are conducted.^[22]^ When there are significant differences among the 3 approaches in a parameter, rankogram will be drawn to shown the probability of best treatment.^[21]^

#### 2.8.1 Publication bias

According to Cochrane Handbook, when enough original studies are included (generally >10 trials), publication bias analysis will be performed through funnel plot.^[23]^ Symmetrical funnel plot indicates low publication bias, otherwise high risk.

## Author contributions

**Data curation:** Dongxu Li, Zhao Zhang.

**Formal analysis:** Dongxu Li, Mengsi Li.

**Methodology:** Dongxu Li, Mengsi Li.

**Project administration:** Mengsi Li.

**Supervision:** Mengsi Li.

**Writing – original draft:** Dongxu Li.

**Writing – review & editing:** Dongxu Li, Zhao Zhang, Mengsi Li.

Mengsi Li orcid: 0000-0001-6765-2159.

## Supplementary Material

Supplemental Digital Content
