# Supplementary material for: Comparisons of periventricular device closure, conventional surgical repair, and transcatheter device closure in patients with congenital ventricular septal defects: A Protocol for Systematic Review
Source: Medicine (Baltimore). 2020 Jan 24;99(4):e18901. doi: 10.1097/MD.0000000000018901 (PMC7004670; doi:10.1097/MD.0000000000018901)
Supplement: Supplemental Digital Content [file medi-99-e18901-s001.docx]

Search strategy via PubMed:

(heart septal defects, ventricular[mh] OR ventricular septal defect*[ti] OR intraventricular septal defect*[ti]) AND (closure[ti/ab] OR surg*[ti/ab] OR repair*[ti/ab] OR occluder*[ti/ab] OR minimally invasive[ti/ab] OR transcatheter*[ti/ab] OR percutaneous[ti/ab] OR transthoracic[ti/ab] OR perventricular[ti/ab]) NOT (animals[mh] NOT humans[mh])
